# The Gridlock Between Chronic Cerebral Venous Thrombosis and Dural Arteriovenous Fistulas

**DOI:** 10.7759/cureus.35035

**Published:** 2023-02-15

**Authors:** Sai Siddartha Kosinepalli, Sudha Kiran Das, Gurumurthy B, Kavitha Chittaragi

**Affiliations:** 1 Radiology, Jagadguru Sri Shivarathreeshwara Medical College, Mysuru, IND

**Keywords:** complications, intracerebral hemorrhage, chatgpt, cerebral venous thrombosis, dural arteriovenous fistula

## Abstract

Cerebral venous thrombosis (CVT) is a rare condition that occurs when a blood clot forms in the veins that drain blood from the brain. The incidence of CVT is estimated to be 1.3-1.6 cases per 100,000 adults, with a higher prevalence in females than males. Dural arteriovenous fistulas (DAVFs) are abnormal connections between the dural sinuses and the venous system, which can occur as a complication of CVT. The incidence of DAVFs after a diagnosis of CVT is reported to be between 0.9 and 13%. It is believed that a thrombus in the cerebral venous system causes stagnation of blood flow leading to an increase in venous pressure and causing enlargement of pre-existing physiological arteriovenous shunts or neoangiogenesis, resulting in the development of a DAVF. However, it is difficult to establish a causal relationship between CVT and DAVF as most low-grade DAVFs are asymptomatic and lack evidence. High-grade DAVFs are considered to be a more serious form of the condition, as they are associated with a higher risk of intracerebral hemorrhage (ICH) and other neurological complications. In our case series of a total of two cases, all were diagnosed first with CVT, and later in the follow-up imaging, chronic CVT with dural AV fistula was observed. This highlights the importance of long-term follow-up imaging in patients with CVT to detect any potential complications such as DAVF, especially in high-risk patients, and to ensure the prompt treatment to prevent serious complications.

## Introduction

Cerebral venous thrombosis (CVT) is a rare condition that occurs when an embolus forms in the veins that drain blood from the brain. The incidence of CVT is estimated to be 1.3-1.6 cases per 100,000 adults, with a higher prevalence in females than males [1]. Factors that increase the risk of developing CVT include pregnancy, oral contraceptive use, and certain medical conditions such as cancer, infection, and genetic thrombophilia. Symptoms of CVT can include headaches, seizures, vision changes, and neurological deficits. Early diagnosis and treatment are essential in preventing severe complications and improving outcomes. Dural arteriovenous fistulas (DAVFs) are abnormal connections between the dural sinuses and the venous system, which can occur as a complication of cerebral venous thrombosis (CVT). The incidence of DAVFs after a diagnosis of CVT is reported to be between 0.9 and 13% [2]. DAVFs can cause several symptoms, including headache, seizures, and neurological deficits, and if not treated, can lead to serious complications such as brain hemorrhage and venous hypertension. Treatment options for DAVFs include endovascular embolization, surgical ligation, and radiation therapy. Thrombolysis is done using drugs like low molecular weight heparin, warfarin, etc. Endovascular treatment can be done in heparin-non-responding subjects. A decompressive craniotomy is performed in cases with increased intracranial pressure and brain herniation.

Cognard classification of DAVFs is currently being used for grading the DAVFs. The classification is, I - Venous drainage into dural venous sinus with the antegrade flow; IIa - Venous drainage into dural venous sinus with the retrograde flow; IIb - Venous drainage into dural venous sinus with the antegrade flow and cortical venous reflux; IIa+IIb - Venous drainage into dural venous sinus with the retrograde flow and cortical venous reflux; III - Venous drainage directly into subarachnoid veins (cortical venous reflux only); IV - Type III with venous ectasia of the draining subarachnoid veins. It can be challenging for radiologists to diagnose DAVFs because many of these fistulas are asymptomatic, and a significant proportion of them are low-grade. High-grade DAVFs are considered to be a more serious form of the condition, as they are associated with a higher risk of intracerebral hemorrhage (ICH) and other neurological complications. Additionally, there is a lack of evidence to determine if CVT causes DAVFs or if DAVFs lead to CVT, making it difficult to establish a causal relationship. This can make it challenging for radiologists to distinguish between the two conditions and make an accurate diagnosis. However, advanced imaging techniques, such as MRI and CT, can help to identify the presence of a DAVF, and angiography can confirm the diagnosis. In addition, the treatment of both CVT and DAVF is different, so it is important to have the correct diagnosis. This approach is a standard of care for diagnosing and managing CVT patients, especially in high-risk patients, to detect potential complications such as DAVF and ensure the prompt treatment to prevent serious complications.

Materials and methods

Over the past two years, we have observed two CVT cases in a tertiary care hospital in India. These patients presented with severe headache and giddiness and were initially evaluated with an MRI brain with MR venogram using a 3.0 Tesla MRI scanner with the use of a 32-channel receive-only head coil to look for cerebral venous sinus thrombosis and venous infarcts. The patients were managed conservatively with thrombolytic therapy. Regular follow-up of the patients was done using the same 3.0 Tesla MRI scanner described above to look for any complications of CVT, such as DAVFs. To confirm the presence of a DAVF, digital subtraction angiography using Phillips Allura Digital subtraction angiography was performed by cannulating different carotid vessels with anteroposterior and lateral projections.

## Case presentation

Case 1

**Figure 1:**
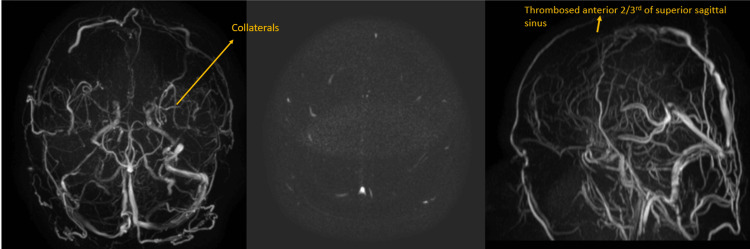
Magnetic resonance venography shows thrombosis of the anterior 2/3rd of the superior sagittal sinus.

**Figure 2:**
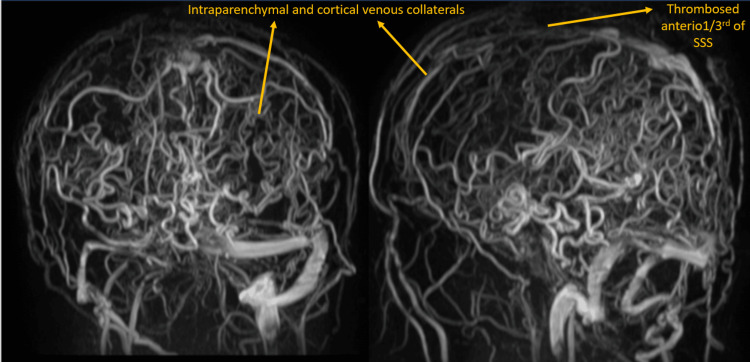
Follow-up MR venogram shows multiple intraparenchymal and cortical venous collaterals.

**Figure 3:**
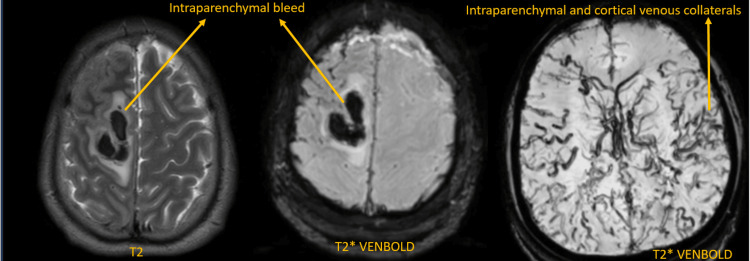
Follow-up MRI in T2W and T2* axial sections shows intraparenchymal bleed in the right frontal lobe. T2W: T2-weighted image.

**Figure 4:**
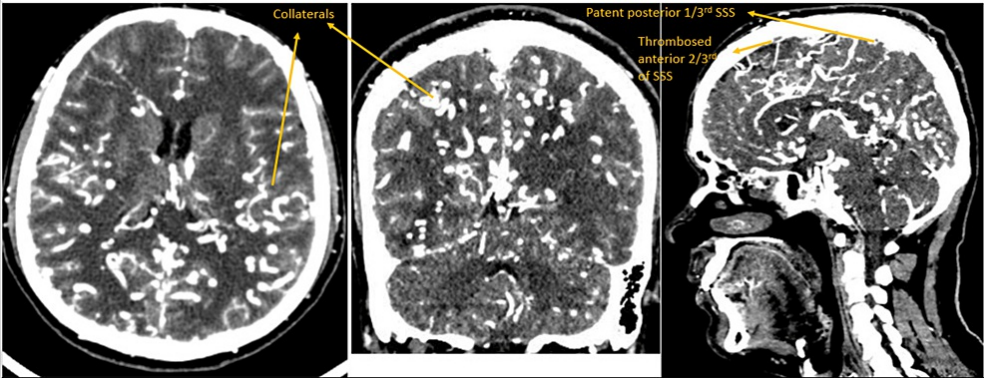
Contrast-enhanced CT head and neck - axial, sagittal, and coronal sections - shows diffuse tortuous cortical, intraparenchymal, and extracranial veins in bilateral cerebral and cerebellar hemispheres suggestive of dural arteriovenous fistula.

**Figure 5:**
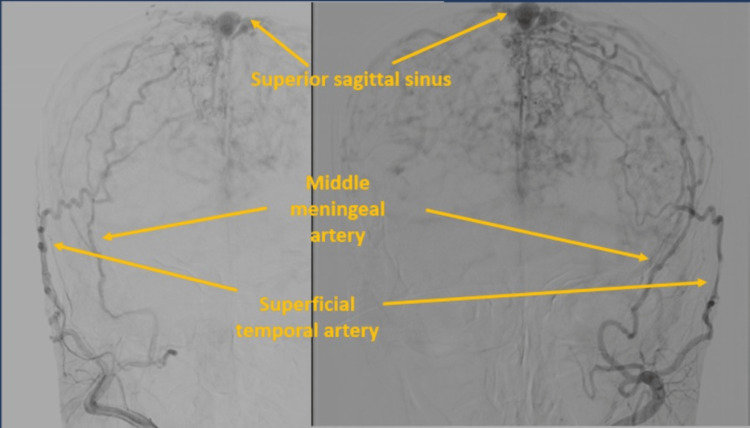
Digital subtraction angiography - canalization of bilateral ECA show superficial temporal and middle meningeal arteries seen feeding the fistula. ECA: External carotid artery.

**Figure 6:**
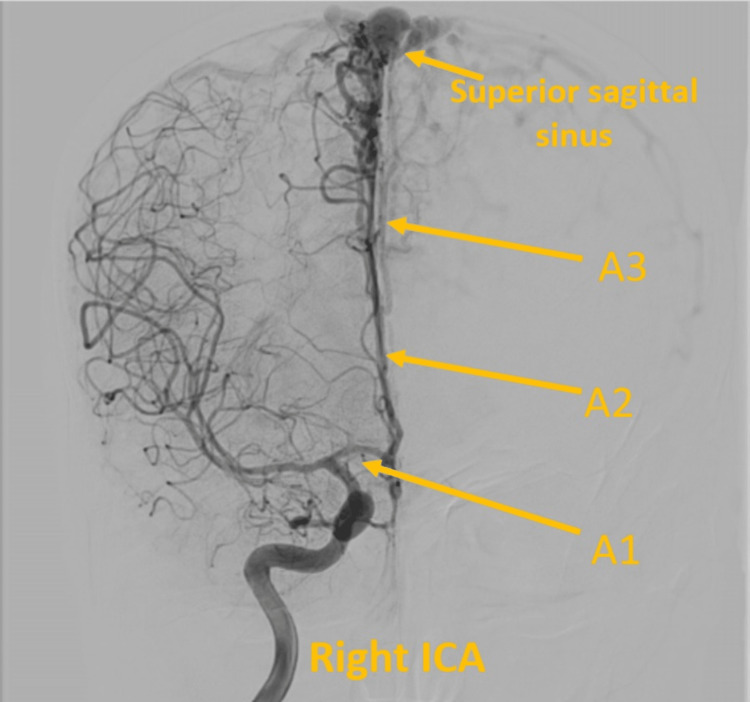
Digital subtraction angiography- right ICA canalization showing pial branches of A2 and A3 of right ACA supplying the fistula. ACA: Anterior cerebral artery; ICA: Interior carotid artery.

A 41-year-old male was evaluated for a presentation of severe headache and giddiness. Routine investigations were normal, but he had slightly elevated homocysteine levels of 17 and low folate levels of 0.6 ng/mL. The patient's initial MRI brain showed thrombosis of the anterior 2/3rds of the superior sagittal sinus, with no intraparenchymal bleed or infarct seen (Figure 1). The patient was managed conservatively with thrombolytics and anticoagulants. However, a follow-up MRI brain with magnetic resonance venography (MRV) one year after the initial diagnosis showed chronic thrombosis of the anterior 2/3rds of the superior sagittal sinus with multiple intraparenchymal and cortical venous collaterals (Figure 2). An intraparenchymal bleed was noted in the right frontal lobe (Figure 3). A CT cerebral angiogram performed one year after the initial diagnosis revealed chronic thrombosis of the superior sagittal sinus with partial recanalization, diffuse tortuous intraparenchymal, and cortical veins in the bilateral cerebral and cerebellar hemispheres, which is suggestive of an arteriovenous malformation (AVM) (Figure 4). Digital subtraction angiography (DSA) performed after one year showed a DAVF at the mid superior sagittal sinus (SSS) with feeding arteries from the internal carotid artery and external carotid artery, severe venous congestion, and cortical venous reflux consistent with a type 2a and b DAVF (Figures 5-6). The patient underwent endovascular embolization of the posterior 1/3rd of the SSS DAVF, and the postoperative period was uneventful. The patient was managed with measures to reduce swelling, steroids, anticoagulants, and other supportive measures. This case highlights the importance of early diagnosis and treatment of CVT and its complications to prevent serious complications such as AVM and DAVF.

Case 2

**Figure 7:**
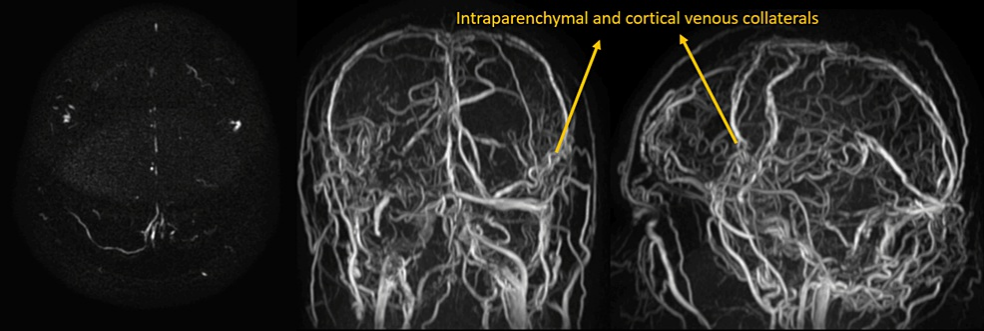
MR venogram shows chronic thrombosis of the superior sagittal sinus with multiple intraparenchymal and cortical collaterals forming a dural arteriovenous fistula.

A 45-year-old male patient presented with complaints of headache and status epilepticus. Routine investigations were normal, with no comorbidities. MRI brain with MRV showed acute hemorrhagic venous infarcts in right parietal-occipital-temporal lobes with cerebral venous thrombosis of posterior-superior dural venous sinuses. The patient was managed conservatively with thrombolytics and anticoagulants. Follow-up MRI brain with MRV after nine months of initial diagnosis showed acute hemorrhagic venous infarcts in right parietal-occipital-temporal lobes and cerebral venous thrombosis of posterosuperior dural venous sinuses with multiple intraparenchymal and cortical venous collaterals suggestive of DAVF (Figure 7).

## Discussion

DAVF constitutes 10-15% of all intracranial arteriovenous malformations [3]. They are more common in females, and symptoms usually develop during middle to late adulthood [4]. Common initiating events include trauma, infection, recent surgery, and dural sinus thrombosis [5]. The most common predisposing factor is venous sinus thrombosis. Hypoxia-induced angiogenesis and reopening of pre-existing channels by venous hypertension are both proposed mechanisms for the development of DAVF following CVT. Hypoxia-induced angiogenesis is a process in which low oxygen levels in the tissue stimulate the growth of new blood vessels as a response to decreased blood flow caused by CVT. The reopening of pre-existing channels by venous hypertension suggests that increased pressure in the venous system caused by CVT may lead to the reopening of previously closed channels within the dura, forming a DAVF [5-6]. Both of these mechanisms may contribute to the development of dAVF following CVT, but the exact pathogenesis is still not fully understood. DAVFs are formed by opening microvascular connections within the dura following venous hypertension, which can lead to direct shunting between arteries and veins. They can be classified based on the type of venous drainage, with type I and II(a) being benign, while the other types have an aggressive course if untreated and carry an 8.1% annual risk of hemorrhage. Endovascular therapy, such as embolization, coiling via a transvenous route, and stenting, are considered first-line treatments for DAVF.

There is a close relationship between cerebral venous sinus thrombosis (CVST) and DAVF, with a reported 39-78% of DAVF patients also suffering from CVST at the same time [7]. Additionally, it cites a study by Pierot L et al. which reported that out of five cases, three patients first developed CVST and then later developed dAVF [8]. This suggests that CVST may be a risk factor or precursor for developing DAVF. Few researchers have suggested a relationship between hypertension of the venous sinus and the development of DAVF, particularly when it is combined with SSS [9]. CVST may increase the risk of developing DAVF by increasing venous sinus pressure; however, it notes that the causal relationship between CVST and DAVF is still controversial. Our case series had CVST first and several years later developed DAVF along with pial AVF, further highlighting the complex relationship between the two conditions.

The limitation of the study is that we lost the follow-up of the patients. DAVF may be a long-term complication of cerebral venous sinus thrombosis (CVST). It may be asymptomatic, leading to an underestimation of its prevalence if follow-up imaging is not performed. After the occurrence of CVST, attention should be paid to determine if DAVF is present through follow-up imaging. Regular follow-up of the patients is required to know whether the DAVF is preceded by cerebrovascular thrombosis or vice-versa. This highlights the importance of regular monitoring and follow-up for patients with CVST to detect and manage any potential complications, such as DAVF, in a timely manner.

## Conclusions

Early diagnosis and treatment of CVT are crucial in order to prevent serious complications such as dural arteriovenous malformations (AVM) and DAVF. These complications can occur due to the formation of DAVFs secondary to CVT. To minimize the risk of these complications, it is important to include follow-up in the treatment protocol for chronic cases of CVT.
